# Association between triglyceride-glucose index and cataract among outpatient US adults

**DOI:** 10.3389/fmed.2025.1523711

**Published:** 2025-05-26

**Authors:** Qi Jin, Jin Huang, Liyun Gao, Jianmin Zhu

**Affiliations:** ^1^The First People’s Hospital of Xiaoshan District, Xiaoshan Affiliated Hospital of Wenzhou Medical University, Zhejiang, China; ^2^National Clinical Research Center for Ocular Diseases, Eye Hospital, Wenzhou Medical University, Wenzhou, China

**Keywords:** cataract, triglyceride-glucose (TyG) index, cross-sectional study, National Health and Nutrition Examination Survey (NHANES), outpatient US adults

## Abstract

**Background:**

Although numerous studies have associated a higher TyG index with various diseases, there is limited research on its potential link to cataract. This study seeks to investigate the relationship between the TyG index and cataract in the outpatient adult population in the United States.

**Methods:**

Our study used NHANES data from the 1999–2008 cycles. We applied weighted multivariate logistic regression to investigate the relationship between the TyG index and cataract in the United States and conducted subgroup analysis to assess the robustness of these associations across different populations.

**Results:**

Among 5,433 adults [2,699 (46.8%) male; 2,734 (53.2%) female], 1,038 (15.4%) had cataract. A fully adjusted model (i.e., model 2) showed that the highest quartile array of TyG index (Quartile IV) was positively associated with a higher risk of cataract among men (OR = 1.63 (1.10–2.43), *p* = 0.016). No difference was found in the female population.

**Conclusion:**

Our research reveals that the highest quartile array of TyG index (Quartile IV) is associated with a higher risk of cataract among men. This suggests that elevated levels of this index may contribute to the likelihood of developing cataracts, emphasizing the need to consider this metabolic parameter when assessing eye health in male participants.

## 1 Introduction

Globally, 94 million people are blind or visually impaired, with cataract being the most common cause of blindness, particularly affecting the elderly (1–4). In the United States, the prevalence rises from 24.4% among individuals aged 40 and older to over 50% for those aged 75 and above, largely attributed to the aging population (5). Nutritional health, alcohol intake, and tobacco use can significantly impact cataract development, which has also been associated with various systemic issues, including diabetes, high blood pressure, and obesity (6, 7). While cataract surgery is effective, economic barriers can limit access, especially in less economically developed countries.

The TyG index, calculated from TG and FPG levels, is a reliable surrogate marker of insulin resistance (8–10). Research indicates that in patients with type 2 diabetes, longer diabetes duration and poor metabolic control, particularly hypercholesterolemia, alongside elevated diastolic blood pressure and reduced kidney function, are significant risk factors for cataract development (11). In addition, a study showed that increased serum levels of LDL-C and TG are independent risk factors for cataract in both male and female participants (12). A study conducted in Saudi Arabia found that in patients with type 2 diabetes, cataract is significantly associated with factors such as age, duration of diabetes, and blood pressure. Notably, the study also revealed that a later onset of diabetes is correlated with a higher incidence of cataract (13). In a related study, it was revealed that diabetic retinopathy significantly increases the risk of developing sight-threatening cataract that require surgery (14). Patients with diabetic cataract show a similar overall bacterial composition to non-diabetic patients but with a significant decrease in beneficial bacteria and an increase in pathogenic strains, indicating a greater susceptibility to infections associated with cataract development (15).

Existing research indicates that both high blood glucose and elevated lipid levels are significant, independent contributors to the development of cataract. The TyG index, calculated from TG and FPG levels, has not been studied in relation to cataract, but it is likely associated with this condition.

## 2 Methods

### 2.1 Data sources and study design

Cataract information was only available in the NHANES cycles from 1999 to 2004 for adults aged 50 and older and in the 2005 to 2008 cycles for those aged 20 and older (16). In this study using data from the 1999–2008 NHANES, adults aged 50 years or older were extracted. All data were analyzed between September and November 2024, and this study followed the STROBE reporting guideline.

**Figure 1:**
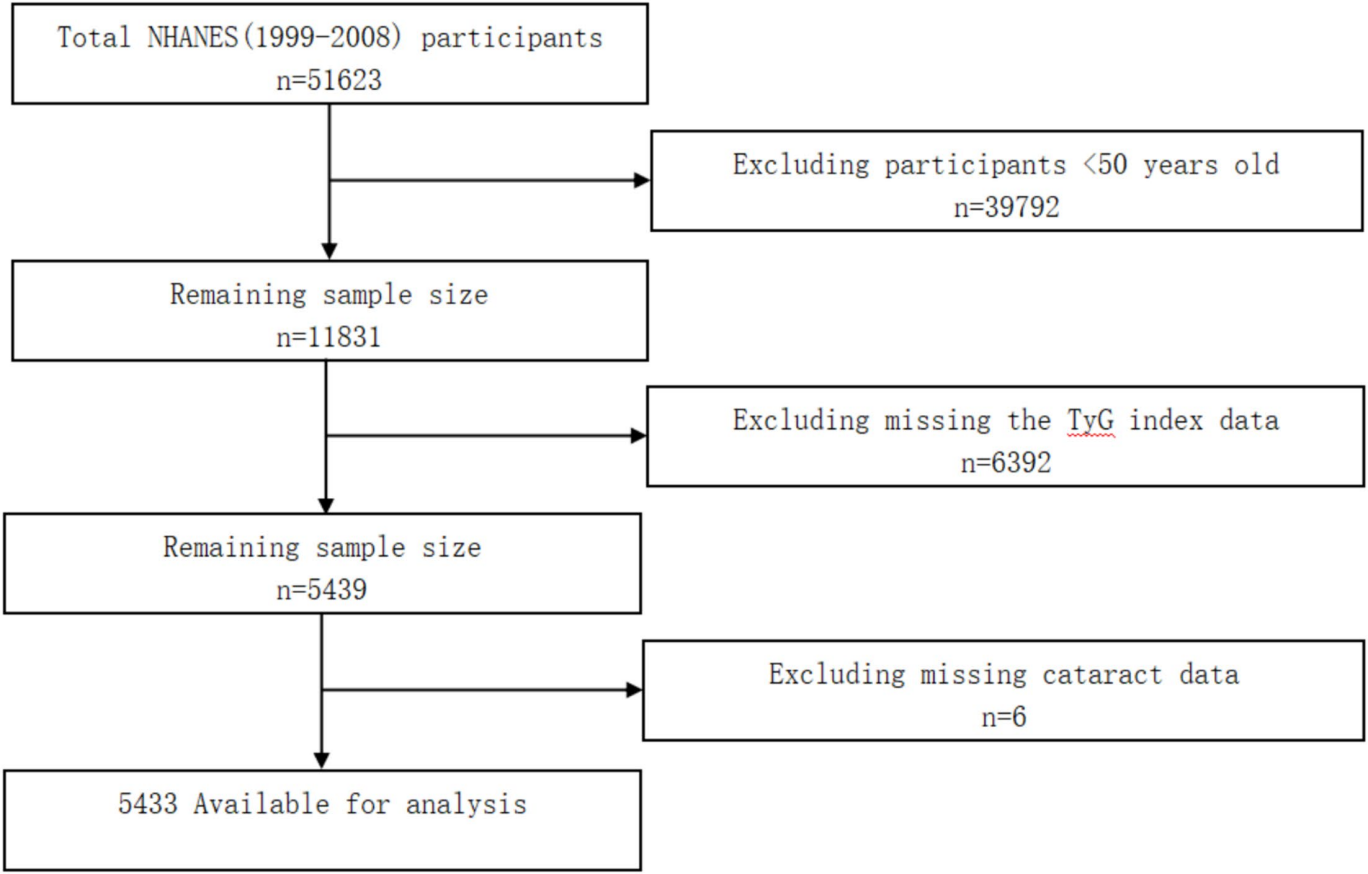
Flow diagram of the screening and enrolment of study participants.

First of all, we identified and selected 11,831 adults aged 50 and older for inclusion in our study. Then, we excluded 6,392 adults who had missing the TyG index data and 6 adults who had missing cataract data. Finally, a total of 5,433 adults were included in the analysis (see Figure 1).

### 2.2 Assessment of TyG index and cataract

The TyG index was calculated as ln [TG × FPG/2] (17–19). Cataract diagnosis in this study was based on the question “Have you ever had a cataract operation?” [NHANES Variable Name: VIQ070 (from 1999–2002 cycles) and VIQ071 (from 2003–2008 cycles)], with response options of “yes” or “no.” A positive response “yes” was considered indicative of a cataract (5, 16, 20).

### 2.3 Covariates

This cross-sectional study examined several potential confounding factors, including sociodemographic variables [age, sex (female/male), race and ethnicity, family income (low/medium/high), educational level, and marital status (married/not married)], NHANES cycles, BMI, alcohol drinking status, and smoking status.

### 2.4 Statistical analysis

Our analysis, which utilized NHANES data from the 1999–2008 cycles, took into account the complex design and applied appropriate weights. Weighting was performed using the NHANES-recommended method, with the fasting subsample weight for the 10-year MEC calculated by dividing the 2-year MEC weight by 5 (21–23).

In the model 1, adjustments were made for sociodemographic factors and NHANES cycles. The model 2 included additional adjustments for BMI, alcohol drinking status, and smoking status. Finally, we performed a subgroup analysis based on sex to examine how it may influence the association between the TyG index and cataract.

Given that the amount of missing data for each variable was minimal, we did not apply any imputation techniques. All statistical analyses were conducted using R.

## 3 Results

As shown in Figure 1, 39,792 adults <50 years old were excluded, and 6,392 adults were excluded for missing the TyG index data. After removing 6 adults missing cataract data, 5,433 adults were included in the analysis (see Figure 1). This study included 5,433 adults, consisting of 2,699 male (46.8%) and 2,734 female (53.2%) participants. In this study, the TyG index was divided into four quartiles (see Table 1), with the highest quartile being more likely to be male (*p* < 0.001).

**Table 1:** Characteristics of participants according to TyG index.

	Participants	TyG index				
Characteristic	Total (N = 5,433)	Quartile I (N = 1,360)	Quartile II (N = 1,354)	Quartile III (N = 1,360)	Quartile IV (N = 1,359)	*p*-value
Age (median [IQR])	61.00 [55.00, 71.00]	60.00 [54.00, 70.00]	62.00 [55.00, 73.00]	63.00 [55.00, 71.00]	61.00 [55.00, 70.00]	0.001
BMI (median [IQR])	27.78 [24.42, 31.84]	25.45 [22.60, 29.37]	27.02 [24.09, 31.20]	28.55 [25.47, 32.58]	29.59 [26.83, 33.60]	<0.001
Sex						<0.001
Male	2,699 (46.8)	657 (42.6)	666 (46.1)	659 (46.4)	717 (52.5)	
Female	2,734 (53.2)	703 (57.4)	688 (53.9)	701 (53.6)	642 (47.5)	
Marital status						0.522
Married	3,191 (65.2)	774 (64.8)	789 (64.1)	824 (67.2)	804 (64.7)	
Not married	2,242 (34.8)	586 (35.2)	565 (35.9)	536 (32.8)	555 (35.3)	
Educational level						<0.001
High school or less	3,223 (49.0)	723 (42.1)	781 (47.5)	807 (49.5)	912 (57.6)	
Some college	1,209 (26.7)	324 (26.7)	300 (27.1)	299 (26.3)	286 (26.6)	
College graduate or higher	988 (24.3)	312 (31.2)	267 (25.4)	251 (24.2)	158 (15.8)	
Race and ethnicity						<0.001
Mexican American	997 (4.2)	144 (2.4)	213 (3.6)	275 (4.7)	365 (6.5)	
Non-Hispanic White	3,076 (79.6)	769 (79.1)	794 (81.1)	783 (80.0)	730 (78.3)	
Non-Hispanic Black	911 (8.1)	361 (12.7)	228 (7.8)	179 (6.1)	143 (5.6)	
Other	449 (8.0)	86 (5.8)	119 (7.5)	123 (9.2)	121 (9.6)	
Family income^a^						<0.001
Low	1,318 (16.6)	304 (14.8)	314 (16.6)	311 (15.2)	389 (20.0)	
Medium	1994 (37.8)	462 (33.6)	492 (36.6)	543 (42.4)	497 (38.8)	
High	1,613 (45.6)	458 (51.6)	426 (46.7)	383 (42.4)	346 (41.2)	
Smoking status^b^						0.051
Never	2,483 (44.5)	657 (47.6)	648 (45.2)	612 (45.3)	566 (39.7)	
Former	2074 (38.5)	501 (37.2)	487 (37.0)	528 (37.6)	558 (42.5)	
Current	867 (16.9)	200 (15.2)	216 (17.8)	218 (17.1)	233 (17.8)	
Alcohol drinker^c^						0.203
Yes	3,622 (67.8)	916 (69.3)	932 (69.7)	875 (66.3)	899 (65.6)	
No	1811 (32.2)	444 (30.7)	422 (30.3)	485 (33.7)	460 (34.4)	
Hypertension						<0.001
Yes	2,781 (48.1)	618 (38.5)	649 (45.8)	714 (50.7)	800 (58.3)	
No	2,652 (51.9)	742 (61.5)	705 (54.2)	646 (49.3)	559 (41.7)	
Hyperlipidemia						<0.001
Yes	2,362 (52.1)	442 (37.7)	551 (48.1)	644 (57.1)	725 (66.6)	
No	3,071 (47.9)	918 (62.3)	803 (51.9)	716 (42.9)	634 (33.4)	
Diabetes mellitus						<0.001
Yes	967 (13.7)	104 (5.7)	143 (7.2)	220 (12.2)	500 (30.8)	
No	4,466 (86.3)	1,256 (94.3)	1,211 (92.8)	1,140 (87.8)	859 (69.2)	
Cataract^d^						0.002
Yes	1,038 (15.4)	241 (13.2)	294 (18.3)	245 (14.2)	258 (15.8)	
No	4,395 (84.6)	1,119 (86.8)	1,060 (81.7)	1,115 (85.8)	1,101 (84.2)	

Data are presented as unweighted number (weighted percentage) unless otherwise indicated.

a Categorized into the following three levels based on the family poverty income ratio: low income (≤1.3), medium income (>1.3 to 3.5), and high income (>3.5).

b We categorized smoking status into the following three groups: never smoked (smoked <100 cigarettes in life), former smoker (smoked at least 100 cigarettes in life but has quit), and current smoker (smoked at least 100 cigarettes in life and is now still smoking).

c Determined by answering the following question: “In any 1 year, have you had at least 12 drinks of any type of alcoholic beverage?”

d Cataract operation was determined by asking participants the question, “Have you ever had a cataract operation?” (VIQ070: 1999–2002; VIQ071: 2003–2008), with answers “yes” or “no.” If the answer was “yes,” the participant was diagnosed with a cataract.

There was no significant difference between the TyG index and cataract in the fully adjusted model (Model 2, see Table 2). In the male subgroup, it demonstrated that the highest quartile array of TyG index (Quartile IV) was associated with a higher risk of cataract [OR = 1.63 (1.10–2.43), *p* = 0.016]. No difference was found in the female subgroup.

**Table 2:** Association between TyG index and cataract.

	TyG index			
	Quartile I	Quartile II (OR, 95% CI, *p*-value)	Quartile III (OR, 95% CI, *p*-value)	Quartile IV(OR, 95% CI, *p*-value)
Overall				
Crude model^a^	1.0 (Reference)	1.47 (1.20–1.80) *p* < 0.001	1.09 (0.87–1.35) *p* = 0.454	1.23 (1.01–1.49) *p* = 0.036
Model 1^b^	1.0 (Reference)	1.39 (1.06–1.83) *p* = 0.019	1.05 (0.79–1.40) *p* = 0.737	1.33 (1.03–1.71) *p* = 0.027
Model 2^c^	1.0 (Reference)	1.32 (1.01–1.73) *p* = 0.041	0.98 (0.73–1.32) *p* = 0.897	1.22 (0.94–1.57) *p* = 0.128
Sex				
Female				
Crude model	1.0 (Reference)	1.62 (1.25–2.10) *p* < 0.001	1.19 (0.91–1.56) *p* = 0.209	1.42 (1.10–1.83) *p* = 0.008
Model 1	1.0 (Reference)	1.26 (0.89–1.78) *p* = 0.183	0.89 (0.63–1.26) *p* = 0.506	1.09 (0.76–1.58) *p* = 0.638
Model 2	1.0 (Reference)	1.14 (0.82–1.59) *p* = 0.425	0.79 (0.58–1.09) *p* = 0.151	0.90 (0.64–1.27) *p* = 0.550
Male				
Crude model	1.0 (Reference)	1.30 (0.94–1.80) *p* = 0.111	0.97 (0.68–1.40) *p* = 0.885	1.09 (0.79–1.50) *p* = 0.593
Model 1	1.0 (Reference)	1.57 (1.07–2.31) *p* = 0.021	1.34 (0.88–2.03) *p* = 0.170	1.71 (1.20–2.43) *p* = 0.003
Model 2	1.0 (Reference)	1.53 (1.03–2.29) *p* = 0.038	1.24 (0.77–1.99) *p* = 0.369	1.63 (1.10–2.43) *p* = 0.016

CI, confidence interval; OR, odds ratio; TyG index, triglyceride-glucose index.

a Crude model: adjusted for none.

b Adjusted for sociodemographic variables (age, sex, race and ethnicity, family income, educational level, and marital status) and NHANES cycles.

c Adjusted for sociodemographic variables, NHANES cycles, BMI, alcohol drinking status, and smoking status.

## 4 Discussion

Cataract is a condition characterized by the gradual clouding of the eye’s natural lens. This clouding interferes with the passage of light to the retina, leading to blurry vision. Existing research indicates that both high blood glucose and elevated lipid levels are significant, independent contributors to the development of cataract. The TyG index, calculated from TG and FPG levels, is a reliable surrogate marker of insulin resistance. Some studies have shown that the higher TyG index levels are significantly associated with the presence and severity of diabetic retinopathy (24–26), but there is a lack of large-scale studies that directly explore the relationship between the TyG index and cataract. In this study of 5,433 participants, we found that the highest quartile array of TyG index is linked to an increased risk of cataract in men. Therefore, the TyG index, with its low cost and non-invasive methodology, could serve as a valuable biomarker for cataract in this population.

This study examined the relationship between the TyG index and cataract in men, finding that the highest quartile array of TyG index (Quartile IV) was associated with a higher risk of cataract in men. Biological, behavioral, and sociological factors likely underlie the sex-specific association between the TyG index and cataract risk. Anatomically, men’s central obesity promotes insulin resistance and raises the TyG index, increasing cataract risk, while pre-menopausal women’s estrogen boosts insulin sensitivity and mitigates the metabolic harm of high TyG levels, and post-menopausal women experience less drastic body fat changes. Behaviorally, men’s higher smoking and alcohol use disrupt metabolism and amplify the impact of a high TyG index, compounded by greater exposure to environmental stressors from physical labor and outdoor activities, whereas women’s emphasis on health management preserves metabolic health and weakens this link. Socially, men’s greater economic stress dysregulates metabolism and heightens risk when TyG is elevated, while women’s proactive health-seeking behavior allows for earlier intervention, reducing the association between the TyG index and cataract development. This suggests that lifestyle changes, such as lowering blood glucose and lipid levels, could be beneficial for individuals with a high TyG index.

Based on our findings, the TyG index appears to be a promising biomarker that could be integrated into broader cataract risk assessments, particularly in high-risk populations, such as those with diabetes or metabolic syndrome. By incorporating the TyG index into routine risk evaluations, healthcare providers could gain valuable insights into an individual’s likelihood of developing cataract, enabling more targeted and personalized approaches to prevention. Routine screening may involve measuring the TyG index alongside other established risk factors for cataract formation, such as age, family history, alcohol, smoking, and UVB exposure.

For individuals with an elevated TyG index, early interventions could focus on lifestyle changes that have been shown to positively impact metabolic health. This might include dietary modifications—such as increasing the intake of antioxidant-rich foods, reducing the consumption of processed sugars, and maintaining a balanced intake of healthy fats—and encouraging regular physical activity to improve insulin sensitivity. For those at even higher risk, medical interventions could be considered, possibly including pharmacological treatments to manage blood sugar and lipid levels.

In addition, increased frequency of eye exams could be recommended for individuals with a high TyG index as regular monitoring would allow for the early detection of cataract development and timely intervention.

## 5 Conclusion

In this study involving 5,433 adults from the 1999–2008 NHANES cycles, we found that the highest quartile array of TyG index (Quartile IV) was associated with a higher risk of cataract in men. It provides important insights into the association between the TyG index and cataract among US adults. Promoting lifestyle changes, such as lowering blood glucose and lipid levels, could be beneficial for individuals with a high TyG index.

## Data Availability

Publicly available datasets were analyzed in this study. This data can be found here: https://www.cdc.gov/nchs/nhanes/.
